# Impact of Quadriceps and Hip Abductor Strengthening on Knee Joint Biomechanics During Gait After Schatzker Type III Tibial Plateau Fracture: A Case Study

**DOI:** 10.7759/cureus.78092

**Published:** 2025-01-27

**Authors:** Satoshi Hakukawa, Kento Kashiwagura, Honami Kishimoto, Akane Saito, Kazuya Fukae

**Affiliations:** 1 Department of Rehabilitation and Care, Reiwa Rehabilitation Hospital, Chiba, JPN; 2 Department of Rehabilitation, Kiminomori Orthopedic Clinic, Chiba, JPN; 3 Department of Rehabilitation and Care, Kiminomori Rehabilitation Hospital, Chiba, JPN

**Keywords:** anterior cruciate ligament injury, gait analysis, lower extremity trauma, musculoskeletal rehabilitation, tibia plateau fracture

## Abstract

Reducing knee joint load during gait in patients after tibial plateau fracture remains a long-term challenge, and effective interventions have not yet been established. This case report involved a 52-year-old female patient, three months after surgery for a tibial plateau fracture accompanied by an anterior cruciate ligament (ACL) avulsion injury. A self-exercise given by the therapist aimed at strengthening both knee extensor and hip abductor muscles was implemented to improve knee joint load during gait. The intervention followed a withdrawal and reversal design (BAB) over two 6-week phases, with weekly strength measurements and assessments of knee flexion angle, performance tests, and three-dimensional gait analysis conducted at the end of each phase. During the intervention period, an increase in knee flexion moment and a decrease in knee adduction moment were observed, indicating improvements in knee joint load. These results underscore the importance of combined knee extensor and hip abductor strengthening exercises.

## Introduction

Tibial plateau fractures are known to be one of the orthopedic conditions that occur as a result of high-energy trauma, such as traffic accidents or sports injuries [1]. Due to the complex mechanical loads involved, these injuries often involve not only isolated fractures but also damage to knee ligaments, meniscus, and articular cartilage [2]. Moreover, tibial plateau fractures generally require postoperative immobilization in knee extension, and depending on the severity of the fracture, rehabilitation protocols often include periods of weight-bearing restrictions. Consequently, adequate recovery of muscle strength is often not achieved, leading to persistent gait instability in the long term. This increases the risk of progressive knee osteoarthritis (OA), often necessitating total knee arthroplasty in the future, highlighting a critical issue in rehabilitation [3].

In rehabilitation aimed at preventing the progression of knee OA, controlling the knee adduction moment (KAM), a key indicator of knee joint load, is considered crucial. KAM is a mechanical factor that increases with the severity of knee OA. Both the magnitude of peak KAM during early stance and the cumulative KAM load (KAM impulse) throughout the stance phase are known to predict the risk of knee OA progression [4,5]. However, the effectiveness of rehabilitation specifically targeting KAM control in tibial plateau fractures with associated high-energy knee trauma remains largely unexplored.

This study aimed to investigate the effects of a self-managed exercise program designed to strengthen both knee extensor and hip abductor muscles on knee joint loading in a patient who had undergone surgery for a tibial plateau fracture with anterior cruciate ligament (ACL) attachment injury, followed by a three-month hospital stay and subsequent discharge to home.

## Case presentation

**Figure 1:**
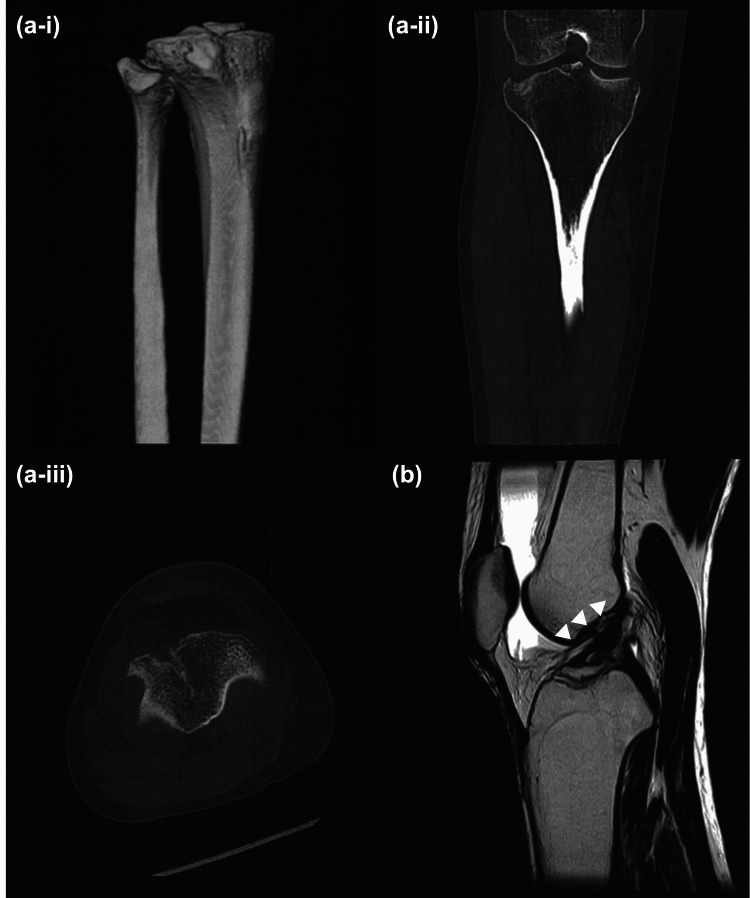
Radiological findings of the tibial plateau fracture (a) Computed tomography (CT) images at the time of injury: (a-i): Three-dimensional CT. (a-ii): Coronal plane. (a-iii): Transverse plane. (b) Magnetic resonance imaging (MRI) image at the time of injury: Suspicion of the anterior cruciate ligament injury.

Written informed consent was obtained from the patient for the publication of this case report. The patient was a 52-year-old female office worker (height: 1.62 m, weight: 46.5 kg, BMI: 17.7 kg/m²) with no significant medical history. In 2024, the patient sustained a right tibial plateau fracture in a traffic accident and was transported to the hospital via emergency services. The fracture involved the displacement of the lateral condyle and extended to the intercondylar area of the tibia, accompanied by an ACL avulsion injury (Figure 1).

**Figure 2:**
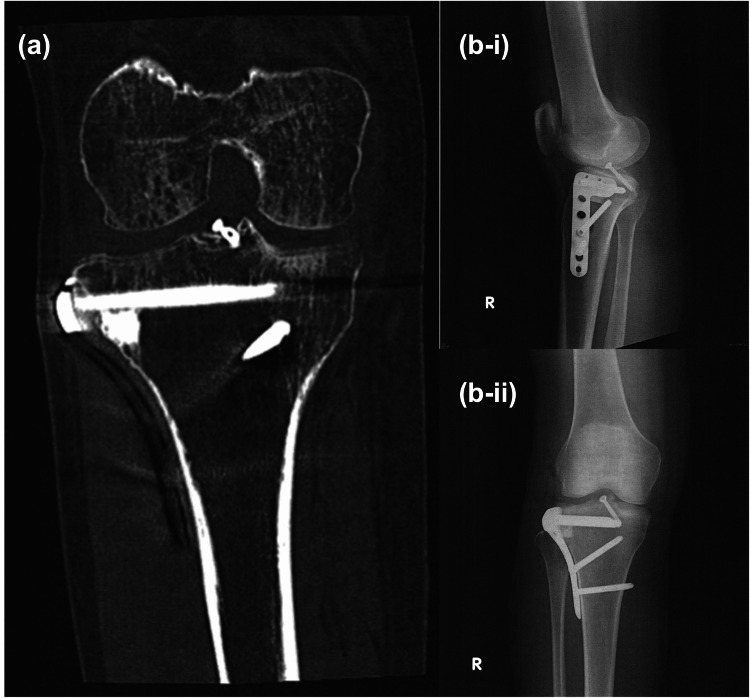
Radiological findings of the tibial plateau fracture after surgery (a) The coronal plane of computed tomography (CT) image after surgery. (b-i) Lateral X-ray findings. (b-ii) Frontal X-ray findings.

Based on the Schatzker classification, the fracture was categorized as Type III. Six days after the injury, the patient underwent open reduction and internal fixation (ORIF) with a plate and screws (Figure 2).

The rehabilitation protocol initially involved non-weight-bearing with the knee immobilized in full extension using a knee brace for the first four weeks postoperatively. This approach aimed to maintain joint surface stability and prevent anterior tibial translation due to the accompanying ACL injury. From the fourth week onward, the knee brace was removed, but the non-weight-bearing status was maintained, and the knee flexion range of motion was restricted to 90°. After the sixth week, range-of-motion restrictions were lifted, and a stepwise weight-bearing protocol was introduced. Specifically, the load was gradually increased every two weeks, progressing from one-third weight-bearing to two-thirds weight-bearing, and ultimately to full weight-bearing.

The patient was transferred to a rehabilitation hospital 19 days postoperatively and discharged 78 days after surgery, at which point she returned to work. Following discharge, the patient commenced outpatient rehabilitation twice a week, with each session lasting 40 minutes. The outpatient rehabilitation program primarily focused on stretching and massage of the periarticular muscles of the knee joint, range-of-motion exercises, and exercise therapy emphasizing open kinetic chain movements. In her daily life, the patient was independently ambulatory without experiencing pain in the operated limb; however, a 10-degree extension lag in the knee joint was observed.

Study design

This study adopted a withdrawal and reversal design (BAB). In Phase A, only outpatient rehabilitation was provided, while in Phase B, outpatient rehabilitation was combined with a self-managed exercise program, with each phase lasting six weeks. Isometric strength of knee extensors and hip abductors was measured weekly. Outpatient rehabilitation was conducted by the same physical therapist with 10 years of clinical experience. At the end of each phase, the following evaluations were performed: functional performance assessments, patient-reported outcome measures (PROMs), and three-dimensional gait analysis.

**Figure 3:**
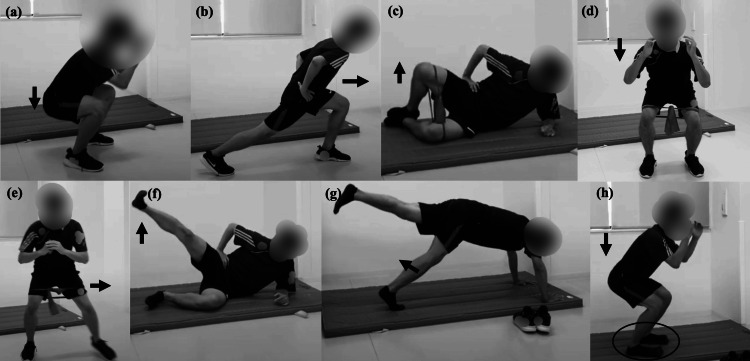
Exercise program (a) Deep squat, (b) front lunge, (c) clamshell, (d) half squat, (e) half squat with a resistance band, (f) side leg raise, (g) single leg squat in a quadruped position, (h) half squat using a slant board.

The self-managed exercise program conducted during the B phase consisted of eight types of exercises, each performed for 30 seconds with 45 seconds of rest between them, organized into two 10-minute sessions. The exercises included deep squat, front lunge, side leg raise, clamshell using a resistance band, half squat with a resistance band, side step abduction with a resistance band, single leg squat in a quadruped position, and half squat using a slant board (Figure 3).

Considering the impact of extension lag in the operated knee joint, the program was designed to avoid excessive loading on the knee joint in the terminal extension range. The program was performed bilaterally, one set for each side, and the participants were instructed to perform the exercises at least three times per week. Instructional videos were shared with the patient via social media.

Assessment and analysis

Knee extensor and hip abductor muscle strength were measured weekly using a handheld dynamometer (HHD; μ-Tas F-One, ANIMA Inc., Tokyo, Japan). Functional performance assessments included measurements of knee joint angles, the 6-minute walk test (6MWT), and the single-leg standing test (SLST). Knee joint angles were measured in the supine position for active and passive flexion using markers placed on the lateral malleolus, fibular head, and lateral femoral epicondyle. These were analyzed using ImageJ software version 1.54k (Open source, Bethesda, MD).

**Figure 4:**
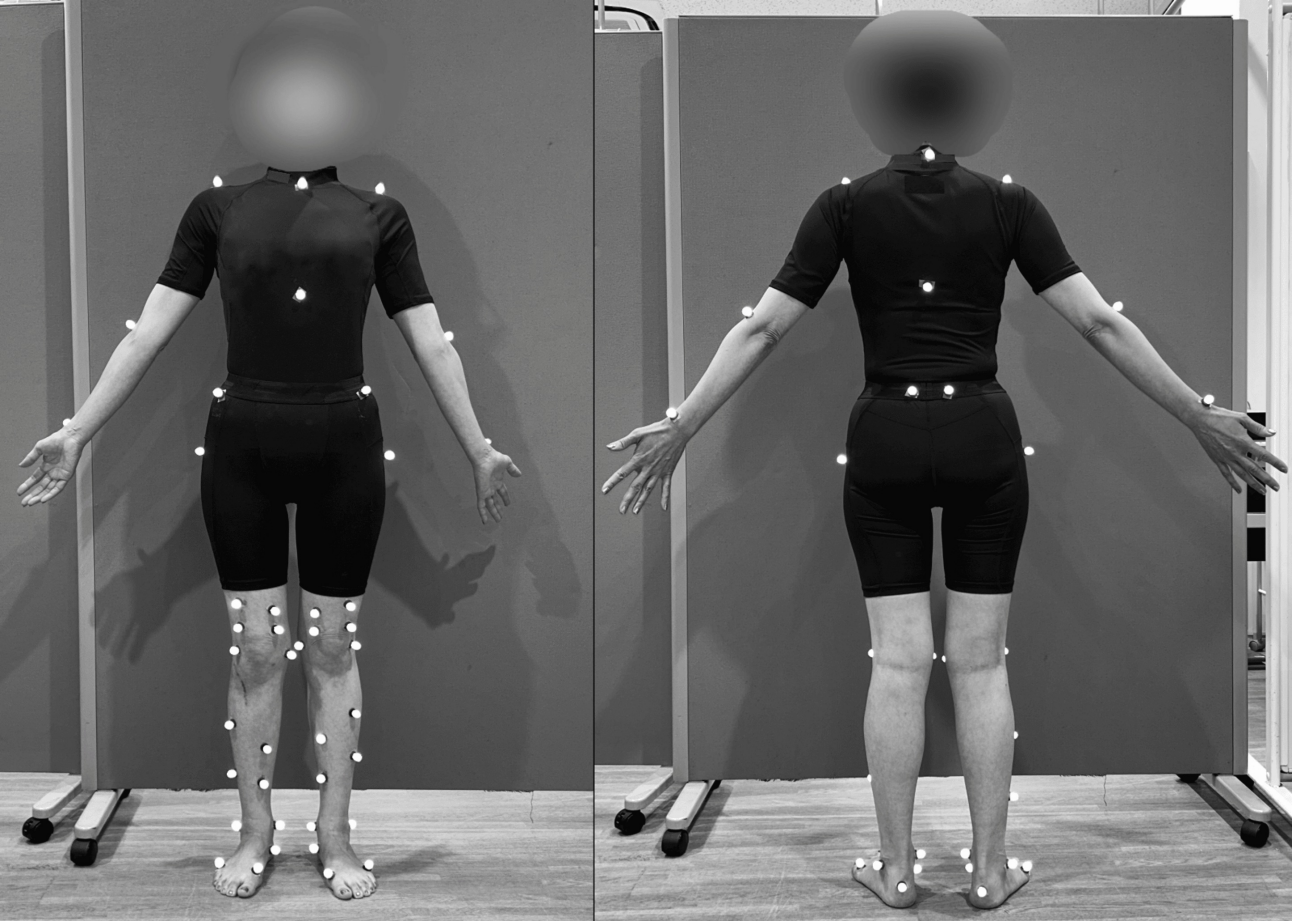
Placement of reflective markers Reflective markers were placed on the following anatomical landmarks: the acromion, manubrium, xiphoid process, anterior superior iliac spine, posterior superior iliac spine, greater trochanter, thigh, medial and lateral femoral epicondyles, shank, medial and lateral malleoli, navicular bone, calcaneus, first and fifth metatarsal heads, lateral epicondyle of the humerus, and radial styloid process.

PROMs were evaluated using the Knee Injury and Osteoarthritis Outcome Score (KOOS) [6], Western Ontario MacMaster University Osteoarthritis Index (WOMAC) [7], and International Physical Activity Questionnaire (IPAQ) [8]. Three-dimensional gait analysis was performed using a motion capture system with 13 infrared cameras and two force plates (VICON Nexus2, Vicon Motion Systems Ltd, Oxford, UK). The task involved level ground walking at a comfortable speed, wearing a specialized suit with 48 reflective markers attached to the body (Figure 4).

Knee extensor strength and hip abductor strength were evaluated to assess the effects of the intervention by visually comparing the trends across phases using graphs. Celeration lines were plotted in the figures to illustrate the trends and compare the changes between phases. Additionally, the percentage of nonoverlapping data (PND) was calculated for the baseline and B1 phases. For the A and B2 phases, the analysis included both PND and the standardized normal difference (SND).

Results

The self-managed exercise program was performed an average of three times per week.

**Figure 5:**
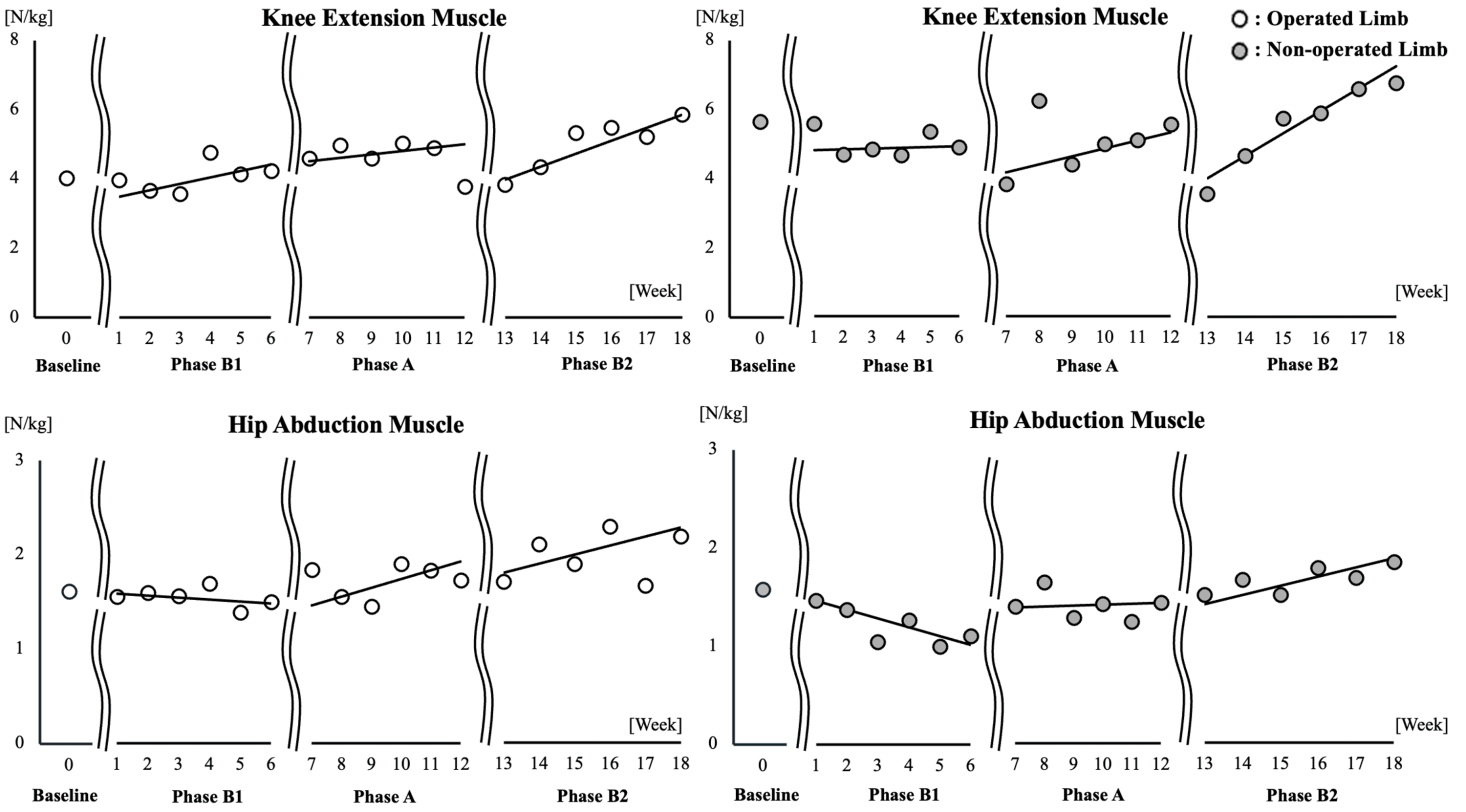
Temporal changes in knee extensor strength and hip abductor strength Weekly strength measurements for each phase were plotted, and celeration lines were created to illustrate the trends in the data.

Figure 5 and Table 1* *present the trends in knee extensor strength and hip abductor strength across phases.

**Table 1:** Changes in knee extensor strength and hip abductor strength Mean values of knee extensor strength (N/kg) and hip abductor strength (N/kg) for the operated and non-operated limbs across each phase are presented. Additionally, the PND was calculated between the baseline and B1 phases, and between the A and B2 phases, while the SND was calculated between the A and B2 phases. Median (range) PND: percentage of nonoverlapping data; SND: standardized normal difference.

Variables, N/kg		Phase						
		Baseline	B1	PND, %	A	B2	PND, %	SND
Operated limb	Knee extension muscle	4.02	4.11 (3.61-4.83)	66.6	4.81 (3.82-5.10)	5.35 (3.89-5.94)	66.6	0.81
	Hip abduction muscle	1.61	1.61 (1.44-1.76)	66.6	1.84 (1.50-1.97)	1.98 (1.73-2.37)	66.6	1.41
Non-operated limb	Knee extension muscle	5.65	4.93 (4.72-5.65)	16.6	5.10 (3.88-6.32)	5.90 (3.61-6.87)	33.3	0.62
	Hip abduction muscle	1.58	1.27 (1.07-1.58)	16.6	1.52 (1.34-1.77)	1.80 (1.62-1.98)	66.6	1.85

In the baseline phase, knee extensor strength was reduced on the operated limb, while hip abductor strength was comparable between the operated and non-operated limbs. During Phase B1, knee extensor strength on the operated limb showed an improving trend, but no significant improvement was observed in the hip abductor strength. In Phase A, knee extensor strength on the operated limb showed a declining trend, while hip abductor strength remained stable. In Phase B2, both knee extensor and hip abductor strength increased bilaterally. The PND showed a large effect size of 0.8 or greater for knee extensor strength and hip abductor strength in the operated limb, as well as for hip abductor strength in the non-operated limb.

 The results of functional evaluations are presented in Table 2.

**Table 2:** Results of physical function assessment The passive and active ROM for knee extension and flexion of the operated limb, the 6MWT, and the SLST results are presented for each phase. Measurements are shown for the Baseline, B1, A, and B2 phases. ROM: range of motion; 6MWT: 6-minute walk test; SLST: single-leg stand-up test.

Variables	Phase			
	Baseline	B1	A	B2
Operated knee passive extension ROM, degrees	0	0	0	0
Operated knee active extension ROM, degrees	0	0	0	0
Operated knee passive flexion ROM, degrees	145.5	155.9	153.6	155.0
Operated knee active flexion ROM, degrees	138.2	149.2	151.0	153.2
6MWT, m	590	655	735	720
Possible SLST on the operated limb, cm	55	45	45	45
Possible SLST on the non-operated limb, cm	50	40	40	45

Regarding knee flexion angles, passive flexion angles showed no significant changes after Phase B1, but active flexion angles improved in all phases. Knee extension angles consistently allowed full extension from the baseline phase onward. The 6MWT showed an increase in walking distance across all phases, with the greatest distance recorded in Phase A. The SLST improved on the operated limb during Phases B1 and B2; however, no notable improvements were observed bilaterally after Phase B1.

The PROM results are shown in Table 3.

**Table 3:** Results of PROMs The results of PROMs for each phase are presented. Evaluation items include the subscales of the KOOS: Symptoms, Pain, and Activities of Daily Living; the WOMAC; and the IPAQ. Measurements are shown for the baseline, B1, A, and B2 phases. KOOS: Knee injury and Osteoarthritis Outcome Score; WOMAC: Western Ontario MacMaster University Osteoarthritis Index; IPAQ: International Physical Activity Questionnaire; PROMs: patient-reported outcome measures.

Variables		Phase			
		Baseline	B1	A	B2
KOOS, number	Symptoms	60.7	60.7	60.7	64.3
	Pain	91.7	88.9	83.3	91.7
	Activities of daily living	89.7	89.7	95.6	97.1
	Sports and recreation function	5.0	30.0	25.0	35.0
	Knee-related quality of life	31.3	31.3	37.5	50.0
WOMAC, number	Pain	0	0	2	0
	Stiffness	2	3	2	1
	Function	5	5	4	3
IPAQ, METs	Vigorous physical activity	0	0	0	0
	Moderate physical activity	0	0	0	0
	Walking	0	264	297	528
	Total METs	0	264	297	528

KOOS, WOMAC, and IPAQ scores were most favorable during Phase B2. KOOS showed significant improvements in the sports and recreation function and knee-related quality-of-life domains compared to the baseline phase. While vigorous and moderate physical activity were not reported in IPAQ, the walking category showed improvements over time.

**Figure 6:**
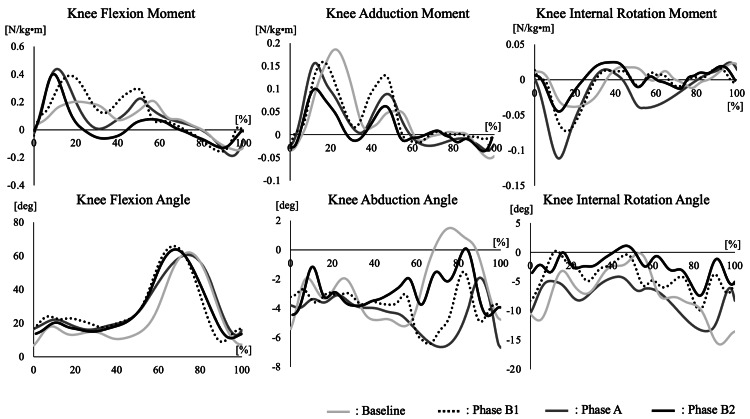
Knee joint kinematics in gait analysis The three-dimensional joint moments and joint angles of the knee during one gait cycle for each phase are presented. The joint moments were normalized to body weight and height.

 The motion analysis results are shown in Figure 6 and Table 4.

**Table 4:** Results of knee joint moment The results of knee joint moments for each phase are presented. Evaluation items include the KAM, KFM, KIRM, and the KAM impulse. Measurements are shown for the baseline, B1, A, and B2 phases. All items were normalized by body weight and height. KAM: knee adduction moment; KFM: knee flexion moment; KIRM: knee internal rotation moment.

Variables	Phase			
	Baseline	B1	A	B2
First peak of KAM, N/kgm	0.186	0.159	0.156	0.099
Second peak of KAM, N/kgm	0.052	0.130	0.089	0.062
First peak of KFM, N/kgm	0.203	0.391	0.439	0.400
Second peak of KFM, N/kgm	0.210	0.297	0.226	0.076
KAM impulse, Ns/kgm	0.033	0.042	0.026	0.014
First trough of KIRM, N/kgm	-0.038	-0.073	-0.111	-0.045
Second peak of KIRM, N/kgm	0.024	0.014	0.015	0.025

The first peak of the KAM was highest in the baseline phase but decreased over time, with a marked reduction observed in Phase B2. KAM impulse also showed a substantial reduction in Phase B2. Conversely, the first peak of the KFM was highest during Phase B2. Knee internal rotation moment showed no significant changes associated with the intervention. Regarding knee joint angles during gait, the baseline phase showed knee extension at the initial stance, but the knee failed to maintain extension, resulting in three flexion occurrences during the gait cycle. Phase B1 exhibited a similar waveform to the baseline phase, although the knee angle during the stance phase was more flexed than in the baseline phase. In Phase A, a "Double Knee Action" waveform was observed, with knee flexion occurring once during stance and once during the swing phase. Phase B2 maintained the double knee action waveform while showing the greatest peak knee flexion angle. The change in knee varus angle during the stance phase was greatest in Phase B2, while the change in internal rotation angle was smallest in Phase B2.

## Discussion

This study evaluated the effects of a self-managed exercise program on reducing knee joint load and improving gait function in a patient after tibial plateau fracture surgery. The results demonstrated that strengthening the quadriceps and hip abductors contributed to improvements in both KFM and KAM.

The patient in this case had a tibial plateau fracture involving the attachment of the ACL. ACL injuries are often associated with two main kinematic abnormalities during gait. The first is a quadriceps avoidance gait, characterized by reduced KFM during the early stance phase due to weakened knee extensor strength [9]. The second is a pivot-shift avoidance gait, in which increased knee flexion angles and reduced internal rotation moments during the terminal stance phase mitigate rotational instability [10]. Gait analysis during the pre-intervention phase in this case revealed a quadriceps avoidance gait, but no evidence of a pivot-shift avoidance gait. This highlights the critical role of quadriceps strengthening in improving gait function. The marked reduction in knee extensor strength, especially on the operated limb, contributed to the observed gait pattern, emphasizing the importance of targeted quadriceps strengthening in addressing this abnormality.

ACL function also influences KAM, as ACL dysfunction has been linked to increased KAM [11]. During the pre-intervention phase, KAM showed a distinct pattern, increasing during the heel contact phase and decreasing during the terminal stance phase. While some studies suggest that quadriceps and gluteus medius strength have a limited impact on peak KAM [12,13], others report that strengthening the gluteus medius can improve the KAM waveform and reduce KAM impulse [14]. The gluteus medius plays a role in reducing medial joint reaction forces during gait [15], and its strengthening may contribute to KAM control, particularly in patients who can maintain knee extension angles post-impact. In this study, KAM impulse increased during the B1 phase, likely due to insufficient improvement in gluteus medius strength. A clear improvement in gluteus medius strength and a significant reduction in KAM impulse were observed during the B2 phase, suggesting that strengthening both the quadriceps and gluteus medius muscles was effective. Despite these improvements, lower limb strength still has room for enhancement. The SLST is a key indicator for locomotive syndrome [16], and the ability to rise from a 40 cm seat is strongly correlated with the presence of knee OA [17]. Therefore, maintaining and improving strength through targeted training is crucial for sustaining and enhancing gait kinematics.

These findings underscore the need for tailored rehabilitation protocols based on fracture type in tibial plateau fractures. Currently, there are no established, effective rehabilitation protocols for tibial plateau fractures [18]. However, it is well known that the gait kinematics of patients with tibial plateau fractures require a considerable amount of time to recover to the level of normal gait kinematics [19,20]. In cases with ligament attachment injuries, like this one, muscle strengthening to compensate for ligament dysfunction must be integrated early in the rehabilitation process. Evaluating the fracture site through imaging and implementing strength training targeted at anticipated kinematic challenges are critical for optimizing recovery.

This study has several limitations. The motion analysis in this study focused solely on level walking under comfortable conditions. As knee joint loads are more pronounced during activities such as stair climbing or walking on uneven surfaces, future studies should incorporate motion analyses under conditions more representative of daily activities. Furthermore, this study did not examine the effects of postoperative lower limb alignment or soft tissue conditions on knee joint loading. Evaluations using full-length lower limb X-rays and ultrasound imaging to assess muscle function might be necessary. Additionally, while the outpatient rehabilitation program avoided explicit muscle-strengthening exercises, its specific content was not strictly standardized. Furthermore, although the intervention program was standardized, it lacked sufficient individualization based on the patient’s condition and recovery stage. Future adjustments to exercise programs tailored to the specific needs and capabilities of patients may further enhance outcomes. Furthermore, conducting gait analysis based on the grade and type of tibial plateau fractures may provide deeper insights into the relationship between these factors and the load on the knee joint.

## Conclusions

A self-managed exercise program was introduced for a patient undergoing outpatient rehabilitation following tibial plateau fracture surgery. The program resulted in improvements in knee extensor and hip abductor strength, reduction of knee joint load, and enhancements in gait kinematics. These findings underscore the importance of early-stage muscle strengthening to maintain and improve gait function post-surgery. Further research involving a larger cohort is needed to validate the effectiveness and optimize the implementation of self-managed exercise programs.
